# Microbiota-derived indole differentially shapes *Entamoeba histolytica* physiology and promotes host-compatible colonization

**DOI:** 10.1371/journal.pntd.0013416

**Published:** 2026-04-13

**Authors:** Eva Zanditenas, Yumiko Saito-Nakano, Meirav Trebicz Geffen, Seiki Kobayashi, Hajime Hisaeda, Tomoyoshi Nozaki, Yuanning Guo, Smruti Mahapatra, Haguy Wolfenson, Serge Ankri

**Affiliations:** 1 Department of Molecular Microbiology, The Bruce and Ruth Rappaport Faculty of Medicine, Technion – Israel Institute of Technology, Haifa, Israel; 2 Department of Parasitology, National Institute of Infectious Diseases, Japan Institute for Health Security, Japan; 3 Department of Biomedical Chemistry, Graduate School of Medicine, The University of Tokyo, Tokyo, Japan; 4 Department of Genetics and Developmental Biology, The Bruce and Ruth Rappaport Faculty of Medicine, Technion – Israel Institute of Technology, Haifa, Israel; University of California San Diego, UNITED STATES OF AMERICA

## Abstract

*Entamoeba histolytica* is a pathogenic amoeba that colonizes the human large intestine and causes amoebiasis. In its natural gut environment, the parasite is exposed to microbiota-derived metabolites, including indole, a tryptophan-derived compound present at millimolar concentrations, whereas laboratory cultures contain negligible levels. We previously showed that bacterial metabolites such as queuine and oxaloacetate modulate parasite stress responses and virulence. Here, we investigated how acute versus long-term exposure to indole shapes *E. histolytica* physiology and host interactions. Trophozoites were gradually adapted to indole over two months. Proteomic profiling compared untreated (WT), acutely indole-exposed (WT + I), and indole-adapted (ADI) trophozoites. Cytoskeletal organization, motility, oxidative stress responses, and colonization capacity were assessed using imaging, functional assays, and a mouse cecum infection model. Host inflammatory responses were evaluated by measuring CXCL1 and lipocalin expression. Acute indole exposure inhibited parasite growth (IC₅₀ = 1.2 mM) and increased cytopathic activity. In contrast, ADI trophozoites displayed reduced cell size, increased F-actin formation, enhanced migration in vitro, and lower cytopathic activity. ADI trophozoites also showed improved survival following oxidative challenge, consistent with enrichment of oxidoreductases and chaperone-related proteins. In vivo, ADI trophozoites colonized the cecum more efficiently than WT or WT + I parasites. Infection with WT + I trophozoites induced higher CXCL1 and lipocalin expression than ADI trophozoites, indicating a stronger host inflammatory response to acutely indole-exposed parasites. These findings identify indole as an ecological cue that initially challenges *E. histolytica* but, upon sustained exposure, promotes adaptive reprogramming toward enhanced stress tolerance and improved host-compatible colonization.

## Author summary

*Entamoeba histolytica* is a protozoan parasite that colonizes the human large intestine and causes amoebiasis, a disease that can range from mild diarrhea to severe invasive colitis and, in some cases, liver abscess. In the gut, the parasite encounters small molecules produced by resident bacteria. One of these molecules is indole, a metabolite derived from tryptophan that is abundant in the intestine but largely absent from standard laboratory cultures. We sought to understand how indole influences parasite behavior and its interaction with the host. We found that sudden exposure to indole stresses the parasite, slowing its growth and increasing its ability to damage host cells, while triggering stronger intestinal inflammation in mice. In contrast, when parasites are gradually exposed to indole over two months, they adapt. Adapted parasites become smaller, move more efficiently, and survive oxidative stress better. They also colonize the intestine more effectively and induce a milder inflammatory response in the host. These findings suggest that indole is not simply toxic or beneficial, but functions as an ecological cue that reshapes parasite physiology over time. Our study highlights how microbiota-derived metabolites can influence parasite fitness and host compatibility, underscoring the importance of incorporating gut-relevant environmental factors when studying intestinal infections.

## Introduction

Amoebiasis, a human disease caused by the protozoan parasite *Entamoeba histolytica*, remains a significant global health challenge, affecting millions of individuals worldwide, particularly in underdeveloped regions with limited access to sanitation and healthcare facilities [1]. This parasite is present in contaminated feces as cysts, a resistant form of the parasite, and once in the host colon, it becomes trophozoites, the infectious form of the parasite. In 10% of the cases, trophozoites become virulent and reach the liver or the lungs, which can lead to the death of the host. This devastating disease is responsible for substantial morbidity and mortality, making it a priority for the development of innovative and effective therapeutic approaches. *E. histolytica* trophozoites reside in the colon, where they are exposed to the gut microbiota, a diverse consortium of microorganisms that plays a fundamental role in maintaining intestinal homeostasis and shaping host immune responses [2].

Emerging research has highlighted the intricate interplay between *E. histolytica* and the gut microbiota, underscoring the importance of the gut microenvironment in shaping the pathogenesis of this parasitic infection (for recent reviews see [3,4]). For instance, cultivating *E. histolytica* with *E. coli* O55 can increase *E. histolytica*’s virulence, which is dependent on the contact between the amoeba and bacteria [5]. Furthermore, *E. histolytica* trophozoites showed greater resistance to oxidative stress (OS) after incubation with *E. coli* O55 [6]. Infection with *E. histolytica* can cause dysbiosis marked by a reduction of *Lactobacillus* and *Bacteroides* and an increase in *Bifidobacterium* [7]. Interactions between *E. histolytica* and the gut microbiota are mediated through a dynamic crosstalk, involving the exchange of metabolites, signaling molecules, and host-derived factors. The gut microbiota can influence the expression of specific virulence-related genes in *E. histolytica*, potentially enhancing its pathogenic potential [8]. Conversely*,* presence of *E. histolytica* modulates the gut microbiota composition, leading to alterations in the microbial community structure and function [7]. Recently, we have discovered that the interaction between *E. histolytica* and gut microbiota is even more complex than previously thought. *E. histolytica* can distinguish between planktonic and biofilm forms of bacteria and degrade these bacterial biofilms through the action of its cysteine proteases (EhCPs) [9].

Although the gut microbiota is increasingly recognized as a modulator of *E. histolytica* behavior, the specific effects of individual microbial metabolites on the parasite’s growth, virulence, and survival remain largely uncharacterized. Oxaloacetate and queuine have emerged as key modulators of the parasite’s response to oxidative stress, contributing to the maintenance of redox balance and cellular homeostasis [10,11]. In parallel, other microbiota-derived metabolites function as signaling molecules capable of reshaping host–microbe–parasite interactions. A prominent example is indole, produced in the colon through bacterial conversion of dietary tryptophan (via tryptophanase) that escapes absorption in the small intestine [12]. Mammalian cells lack the ability to produce indole, making bacterial sources the sole contributors of indole in the human body [13]. While the fecal indole concentration ranges from 0.30 to 6.64 mM, the exact indole concentration in the colon remains unknown but is likely higher [14,15].

In humans, indole and its derivatives play key roles in maintaining intestinal immune homeostasis [16]. Indole reduces inflammation by suppressing IL-8 secretion, inhibiting NF-κB activation, and promoting IL-10 production [17]. Thus, indole contributes to anti-inflammatory and antioxidant defenses in the gut.

*E. histolytica* is exposed to indole produced by bacteria from the microbiota. Interestingly, *E. histolytica* also possesses the enzyme tryptophanase, which catalyzes the conversion of tryptophan into indole and pyruvate, an effective process for ATP production [18]. In the presence of 1 mM tryptophan, *E. histolytica* is able to produce 6.8 ± 0.8 µM of indole after 3 days, which is very low compared to the amount of indole that bacteria such as *E. coli* can produce, up to 600 µM in suspension culture [19,20]. Indole is a well-studied metabolite due to its role in inter- and intra-cellular signaling in bacterial ecosystems. It influences biofilm formation and virulence factors in bacteria [13]. These influences vary between indole-producing and non-producing bacteria. For example, indole enhances biofilm formation in non-indole-producing bacteria such as *Pseudomonas aeruginosa*, whereas it suppresses biofilm formation in indole-producing bacteria like *E. coli* [21].

Our understanding of the effects of indole on parasitic organisms remains limited. In the case of *Cryptosporidium*, higher levels of indole in fecal material have been correlated with reduced severity of diarrhea in patients, suggesting that indole-producing bacteria may impair the parasite’s ability to establish infection in the human intestine, possibly through disruption of mitochondrial functions [22,23]. Similarly, the indole derivative methyl 6-chloro-1H-indole-3-carboxylate induces mitochondrial dysfunction in the free-living amoebae *Acanthamoeba castellanii* and *Acanthamoeba griffini*, triggering reactive oxygen species (ROS) production and programmed cell death [24]. Beyond protozoa, indole also influences bacterial pathogens: for instance, *Porphyromonas gingivalis*, a major contributor to periodontitis, exhibits decreased virulence when its tryptophanase gene is deleted. This loss of indole production alters its proteome and metabolome, leading to diminished expression of virulence factors, reduced biofilm formation, and impaired host interactions [25]. Given that standard components of laboratory media for *E. histolytica* including adult bovine serum are largely indole-deficient ([26,27] and this work), in contrast to the high indole levels present in the colon, and considering its known effects on other microbes and protozoa, we hypothesize that indole may modulate *E. histolytica* physiology. This study explores the impact of indole on *E. histolytica* trophozoites and the underlying molecular mechanisms.

## Methods

### Ethics statement

All animal experiments were approved by the Institutional Animal Care and Use Committee (approval number: 123041) and conducted at the Association for Assessment and Accreditation of Laboratory Animal Care (AAALAC) -accredited National Institute of Infectious Diseases, JIHS, Japan. At the conclusion of the experiments, animals were euthanized by cervical dislocation under isoflurane anesthesia.

### *E. histolytica* culture

*E. histolytica* trophozoites of strain HM-1:IMSS (from Prof. Samudrala Gourinath, Jawaharlal Nehru University, New Delhi, India), were grown at 37 °C in 13 × 100 mm in screw-capped Pyrex glass tubes in Diamond’s TYI S-33 medium, supplemented with bovine serum (Biowest, France), to the exponential phase. Trophozoites were harvested from their growth support by incubating the tubes by tapping the glass tubes, followed by centrifugation (Eppendorf centrifuge 5810R, rotor A-4–62) according to a previously reported protocol [26]. Hamster liver-passaged virulent HM-1:IMSS (v-HM1) was cultured in YIMDHA-S medium supplemented with live *Crithidia fasciculata* at 35°C [28,29].

### HeLa cell cultures

HeLa cells (a kind gift from Dr. Kleinberger, Faculty of Medicine, Technion) were maintained in Dulbecco’s modified Eagle’s medium (DMEM) (Gibco, ThermoFisher Scientific, Inchinnan, Scotland, UK) supplemented with 4 mM l-glutamine, penicillin (100 units ml^−1^), streptomycin (100 μg ml^−1^), and 10% fetal calf serum (Gibco, Thermo Fisher Scientific, Brazil). The media was changed every 2 days. The cultures were grown in 15 by 10 cm plastic tissue culture flasks and maintained in a humidified atmosphere of 5% CO_2_ at 37 °C. The media was changed every 2 days.

### IC_50_

*E. histolytica* trophozoites (1 × 10⁶ cells) in TYI S-33 medium were treated with indole (Sigma-Aldrich, China) at concentrations of 0, 0.5, 1, 2, 3, and 4 mM for 24 hours at 37 °C. Trophozoite viability was assessed using the eosin dye exclusion method (0.1% final concentration) [30].

### Growth rate of *E. histolytica* Trophozoites

A total of 4 × 10^4^ *E. histolytica* trophozoites were grown in a 15 mL tube in TYI-S-33 medium at 37°C. Viable trophozoites were counted after 24 and 48 hours using a previously described protocol [30].

### Adaptation to indole

The concentration of indole in *E. histolytica* trophozoite cultures was gradually increased from 0 to 1.2 mM over a two-month period. Indole concentration was increased stepwise over a two-month period, primarily in 0.5 mM increments every two weeks, followed by a final adjustment to reach 1.2 mM. *E. histolytica* trophozoites adapted to indole are referred to as ADI throughout the manuscript. For reversal experiments, indole was subsequently removed, and ADI trophozoites were maintained in indole-free medium for one month (ADI reverse trophozoites).

### Measurement of cytopathic activity

The cytotoxicity of *E. histolytica* trophozoites toward cultured HeLa cell monolayers was assessed as described in [5]. HeLa cells were used as a standardized and widely adopted in vitro model for evaluating *E. histolytica* cytopathic activity, consistent with numerous previous studies including the seminal work of Bracha and Mirelman [5]. Equal numbers of viable trophozoites (3 × 10⁵ viable cells per well) were incubated with HeLa monolayers in 24-well plates at 37 °C for 30 minutes. The incubation was terminated by placing the plates on ice, and trophozoites were removed by washing with cold PBS containing 1% galactose. Remaining HeLa cells were fixed with 2% (v/v) formaldehyde in PBS and stained with 0.1% methylene blue in 0.1 M borate buffer (pH 8.7). The dye was extracted using 0.1 M HCl, and absorbance was measured at 660 nm with a spectrophotometer.

### Detection of intracellular indole by metabolomics

Intracellular indole levels were measured using three independent biological replicates of 10^7^ cells per experimental condition: wild-type (WT) trophozoites, trophozoites adapted to 1.2 mM indole (ADI), and trophozoites acutely exposed to 1.2 mM indole for 24 h at 37 °C (WT + I). One million trophozoites were harvested by gently tapping the glass tubes, followed by centrifugation at 686 g for 3 minutes at room temperature (RT). Cell pellets were resuspended in phosphate-buffered saline (PBS), transferred to 2 ml homogenizing tubes, and centrifuged again at 686 g for 3 minutes at RT. After removal of PBS, cell pellets were resuspended in 300 µl of ice-cold metabolite extraction solution consisting of methanol (Merck, 106035), acetonitrile (Merck, 100029), and water (5:3:2, v/v/v). For metabolite extraction, the resuspended cell pellets were transferred to CK14 homogenizing tubes containing 1.4 mm ceramic beads (Bertin Corp, P000926-LYSK0-A) and homogenized at 4 °C using a Precellys 24 tissue homogenizer. Homogenization was performed for three cycles of 30 s at 8030 g, with 30 s pauses between cycles to maintain low temperature. Homogenates were centrifuged at 18,000 g for 15 minutes at 4 °C. The resulting clear supernatants were transferred to glass high-performance liquid chromatography (HPLC) vials (Agilent, 8010–0542) and stored at −80 °C until liquid chromatography–tandem mass spectrometry (LC–MS/MS) analysis. Indole levels in TYI-S33 medium were quantified as described above.

### Metabolomics profiling LC-MS analysis

For the detection of polar metabolites, LC–MS–based metabolomics analysis was performed as previously described [31], with minor modifications detailed below. Analyses were carried out using a Thermo Fisher Scientific Vanquish ultra-high-performance liquid chromatography (UHPLC) system coupled to an Exploris 240 Orbitrap mass spectrometer (Thermo Fisher Scientific). Data were acquired at a resolution of 60,000 (at m/z 200) using electrospray ionization in polarity-switching mode, enabling detection of both positive and negative ions over a mass range of 67–1000 m/z. Chromatographic separation was achieved using a ZIC-pHILIC column (SeQuant; 150 × 2.1 mm, 5 µm). Five microliters of biological extracts were injected, and metabolites were separated using a 15-minutes mobile-phase gradient starting at 20% aqueous phase (20 mM ammonium carbonate [Thermo Fisher Scientific, 10785511], adjusted to pH 9.2 with 0.1% of 25% ammonium hydroxide [Thermo Fisher Scientific, 15547049]) and 80% acetonitrile, and ending at 20% acetonitrile. The flow rate was maintained at 0.2 mL/minutes, and the column temperature was set to 45 °C, resulting in a total run time of 27 minutes. All metabolites were detected with a mass accuracy below 5 ppm. Data acquisition was performed using Xcalibur software (Thermo Fisher Scientific).

### Metabolomics data analysis

Skyline V (version 25.1) was used for data analysis. Metabolite retention times on the pHILIC column were predetermined by analysis of an in-house mass spectrometry metabolite library composed of commercially available standards. Metabolite quantification was based on peak areas determined from the exact mass of singly charged ions, such that a larger peak area corresponded to a higher relative abundance of the metabolite. Peak area values for each metabolite were normalized to the protein content (µg) of the corresponding cell lysates. Data visualization was performed using Metabolite AutoPlotter 2.6 (PMID: 32670572) and MetaboAnalyst.

### Preparation of lysate for proteomics

Samples were precipitated overnight at −20 °C by adjusting to a final concentration of 80% ice-cold acetone and subsequently washed three times with cold 80% acetone. Protein pellets were dissolved in 8.5 M urea, 400 mM ammonium bicarbonate, and 10 mM dithiothreitol (DTT). Protein concentration was determined using the Bradford assay. Samples were reduced at 60 °C for 30 minutes, alkylated with 35.2 mM iodoacetamide in 100 mM ammonium bicarbonate for 30 minutes at room temperature in the dark, and digested with modified trypsin (Promega) in 1.5 M urea and 66 mM ammonium bicarbonate overnight at 37 °C using a 1:50 (w/w) enzyme-to-substrate ratio. A second digestion was performed for 4 hours using a 1:100 (w/w) enzyme-to-substrate ratio.

Tryptic peptides were desalted using an Oasis HLB 96-well µElution plate (Waters) or homemade C18 StageTips, dried, and reconstituted in 0.1% formic acid containing 2% acetonitrile.

### Mass spectrometry analysis for proteomics

The resulting peptides were analyzed by LC–MS/MS using an Exploris 480 mass spectrometer (Thermo Fisher Scientific) coupled to a capillary HPLC system (EV-1000, Evosep One, Denmark). Peptides were loaded onto a 15 cm × 150 µm inner diameter column packed with 1.9 µm particles (Performance column EV1137; Evosep, Denmark). Peptide separation was performed using the built-in Xcalibur 15 SPD (88 minutes) method. Mass spectrometry data were acquired in positive ion mode using repeated full MS scans (m/z 380–985, resolution 120,000), followed by data-independent acquisition (DIA) scans with 10 Da isolation windows and 1 m/z overlap at a resolution of 30,000.

### Data analysis

Mass spectrometry data were analyzed using DIA-NN software (version 1.9.2) [32] against the *E. histolytica* (strain ATCC 30459) protein database (UP000001926_2025_02_27.fasta). The minimum peptide length was set to 7 amino acids, with a maximum of one missed cleavage allowed. Carbamidomethylation of cysteine was specified as a fixed modification, and protein N-terminal acetylation was included as a variable modification. Peptide- and protein-level false discovery rates (FDRs) were controlled at 1%. Statistical analysis of protein identification and quantification results was performed using Perseus software (version 1.6.7.0) [33].

### Resistance to hydrogen peroxide (H₂O₂)

To quantify OS resistance, wild-type (WT), acute indole–treated (WT + I), and indole-adapted (ADI) trophozoites were exposed to H₂O₂ (Supelco, Merck, Darmstadt, Germany). Trophozoites (1 × 10⁶ cells) were incubated with 2.5 mM H₂O₂ for 30 minutes at 37 °C. Following treatment, trophozoites were pelleted by centrifugation and resuspended in fresh TYI medium. Cell viability was subsequently assessed using the eosin exclusion assay [30].

### Detection of oxidized proteins in trophozoites

Protein oxidation levels were measured using the OxyBlot Protein Carbonyl Assay Kit (Abcam, Cambridge, UK) according to the manufacturer’s instructions. Wild-type (WT), acute indole–treated (WT + I), and indole-adapted (ADI) trophozoites were exposed to H₂O₂ (2.5 mM for 30 minutes at 37 °C) and subsequently lysed in 1% Nonidet P-40 (NP-40 in PBS) for 15 minutes on ice. Equal amounts of protein (20 µg) were processed using the OxyBlot assay. In this assay, protein carbonyl groups formed during oxidative modification react with 2,4-dinitrophenylhydrazine (DNPH), generating dinitrophenyl (DNP) derivatives that are detected by immunoblotting, thereby providing a quantitative measure of protein oxidation.

### Assessment of protein synthesis through surface sensing of translation (SUnSET)

The SUnSet was performed according to [34]. Briefly, treated trophozoites (2 × 10^6^) were incubated with 10 μg/mL puromycin (Merck, Rehovot, Israel), a structural analog of tyrosyltRNA, for 20 minutes at 37°C. Following this, the trophozoites were lysed using a lysis buffer (10% NP-40, 0.5M E64 (ChemScene, New Jersey, USA), 100 mM PMSF in PBS). The whole proteins were separated on a 10% SDS-PAGE in SDS-PAGE running buffer and subsequently electrotransferred to a nitrocellulose membrane in protein transfer buffer. Loading equivalency was determined by Ponceau-S (Merck, Rehovot, Israel) staining of the membrane before immunostaining. Puromycin incorporation was detected by immunoblotting using a 1:1000 dilution of monoclonal puromycin antibody (12D10 clone, Merck Millipore, Rosh-Ha’ayin, Israel). Following incubation with the primary antibody, the blots were treated with a 1:5000 dilution of secondary antibody (Jackson ImmunoResearch, West Grove, PA, USA) for 1 hour at RT, then developed using enhanced chemiluminescence (WesternBright ECL, Advansta, CA, USA) and photographed with Fusion FX7 Edge Spectra. Protein synthesis quantification was determined from the intensity of the immunoreactive blots (densitometry) using Fiji software [35].

### Actin staining

A total of 1 × 10⁶ *E. histolytica* trophozoites (WT, WT + I, and ADI) were harvested. Trophozoites were resuspended at a density of 1.5 × 10⁵ cells/mL in 1 mL serum-free TYI medium at 37 °C, in the presence or absence of indole (2.5 mM in DMSO), transferred to Ibidi µ-Slide 8-well plates (80826; ibidi GmbH, Gräfelfing, Germany), and allowed to adhere for 1 hour at 37 °C.

Adherent trophozoites were fixed with prewarmed 4% paraformaldehyde (PFA; Electron Microscopy Sciences, Hatfield, PA, USA) at 37 °C for 30 minutes at room temperature (RT). Following fixation, cells were washed three times for 5 minutes with mild agitation in 0.1% Triton X-100 and 0.1% Tween 20 in PBS (PBSTT). To ensure complete permeabilization, samples were incubated in PBSTT for an additional 5 minutes at RT. Cells were then quenched with 50 mM NH₄Cl in PBS for 30 minutes at RT, washed three times, and blocked with 5% bovine serum albumin (BSA; MP Biomedicals, Solon, OH, USA) in PBSTT for 1 hour at RT.

The following day, samples were washed three times with PBSTT and incubated for 4 hours at 4 °C with Alexa Fluor 488 (1:250; Jackson ImmunoResearch, West Grove, PA, USA) and Phalloidin–iFluor 594 (1:500; #ab176757, Abcam, Cambridge, UK), both diluted in 1% BSA in PBSTT. Samples were then washed twice with PBSTT and twice with PBS, mounted using SlowFade Gold Antifade Mountant (#S36937; Invitrogen), and imaged using a Zeiss LSM800 confocal microscope with a 20 × objective. All images were acquired using identical staining procedures and microscope acquisition settings to ensure consistency.

### Quantification of fluorescent intensity

To ensure consistency in planar confocal imaging of fixed samples, all images were acquired using uniform staining protocols and identical microscope acquisition settings. Cell segmentation was performed using the Cellpose-SAM model, which generated precise masks delineating individual cells (https://huggingface.co/spaces/mouseland/cellpose) [36]. These segmentation masks were imported into Fiji for quantitative analysis of fluorescence intensity. Three-dimensional (3D) image analysis was performed using Imaris software (version 10.2). The software automatically detected and counted trophozoites stained with Phalloidin based on the cellular fluorescence signal. For each detected cell, Imaris quantified the Phalloidin fluorescence intensity as well as the cellular volume.

### Transwell migration assays

Transwell migration assays were performed using 5 mm transwell inserts (SPL Insert Hanging, 8 µm pore size, Gyeonggi-do, Republic of Korea) placed in individual wells of 24-well plates, as previously described [37]. WT, WT + I, and ADI trophozoites were harvested and viability was determined using the eosin exclusion assay prior to seeding. Equal numbers of viable trophozoites were then washed once in serum-free Diamond’s TYI-S-33 medium, and 500 µL aliquots containing 3 × 10⁵ viable trophozoites/mL were loaded into the upper chamber of each insert. The plates were placed in anaerobic bags (Mitsubishi Gas Chemical Company, Inc., Tokyo, Japan) and incubated at 37 °C for 3 hours. At the end of the incubation, inserts and media were removed, and trophozoite migration was assessed by counting cells attached to the bottom of the wells. To facilitate detachment, plates were placed on ice for 10 minutes, and viable cells were quantified using the eosin exclusion assay.

### Intra-cecum injection in mice

The liver-passaged v-HM1 strain was maintained by serial passage through the cecum of male C57BL/6NCrSlc mice. Trophozoites isolated from the mouse cecum were monoxenically cultured in YIMDHA-S medium supplemented with *C. fasciculata* [38]. Indole concentration was gradually increased to 1.2 mM to generate ADI trophozoites as described above. Prior to the main challenge, both WT and ADI trophozoites were passed through the mouse cecum for 24 hours to allow recovery and initiation of axenic culture. Cecum-recovered trophozoites were used for the main challenge within 2 weeks of axenic culture.

For the main challenge, six-week-old male C57BL/6NCrSlc mice (Japan SLC, Inc., Shizuoka, Japan) were randomly divided into three groups (n = 5 per group). Mice were anesthetized with isoflurane and surgically injected with 1 × 10⁶ cecum-passed trophozoites in 200 µL of culture medium into the proximal and apical regions of the cecum. The groups were as follows: WT, WT + I, and ADI trophozoites [38,39]. After injection, ceca were blotted, and the peritoneum and skin were sutured. Mice had free access to food and water under standard room temperature and humidity conditions. All mice survived until the scheduled euthanasia. Immunomodulation data results were obtained from one experiment (n = 5 per group).

### Detection and quantification of trophozoites in mouse stool

Body weight was monitored daily for 7 days following challenge. Seven days post-infection, DNA was extracted from 3–5 stool particles using the QIAcube robotic system and the QIAamp Fast DNA Stool Mini Kit (Qiagen, Tokyo, Japan). Infection was assessed by PCR targeting the 18S ribosomal RNA gene of *E. histolytica* (GenBank: AB282658) using the primers EntaF3 (5′-ATCCATGATCGCTATAAGATGCACGAGAG-3′) and EhR-4 (5′-CCATAAACTCAAGATTTCTCTTTAAGTTCTGAACAA-3′). To quantify trophozoite numbers in stool, real-time quantitative PCR was performed with Fast SYBR Green Master Mix (Thermo Fisher Scientific, USA) on a StepOne Plus real-time PCR system (Applied Biosystems, USA) [10].

### Measurement of fecal lipocalin-2 and cecal CXCL1 levels

Lipocalin-2 and CXCL1 (murine IL-8 homolog) levels were measured by enzyme-linked immunosorbent assay (ELISA) as previously described [40]. Briefly, stool samples were homogenized in PBS containing 0.1% (v/v) Tween 20 and centrifuged at 23,000 × g for 10 min at 4°C. The supernatants were analyzed using the Mouse Lipocalin-2/NGAL DuoSet ELISA kit (DY1857, R&D Systems, FujiFilm Wako Pure Chemical Corporation, Japan) according to the manufacturer’s instructions.

For CXCL1 measurement, cecal tissues were collected 24 h after trophozoite infection or sham operation. Resected cecal tissues were homogenized by bead beating in Lysis Buffer I (5 mM HEPES supplemented with 1 × cOmplete Mini Protease Inhibitor Cocktail, Roche, MilliporeSigma, Japan), followed by extraction with 250 μL of Lysis Buffer II (2% Triton X-100 in Lysis Buffer I). Lysates were centrifuged at 13,000 × g for 5 min at 4°C, and supernatants were collected. CXCL1 levels were quantified using a Mouse CXCL1 (GRO-α) ELISA kit (ab155458, Abcam, Japan) according to the manufacturer’s instructions.

### Statistical analysis

Statistical analyses were performed using GraphPad Prism 9.0 (GraphPad Software, Inc., San Diego, CA). For pairwise comparisons, an unpaired two-tailed Student’s t-test was used. For comparisons involving more than two groups, one-way or two-way ANOVA was performed as appropriate. When the overall ANOVA was significant, Fisher’s least significant difference (LSD) post hoc test was applied for pairwise comparisons, as indicated in the corresponding figure legends. For time-course experiments (e.g., fecal lipocalin-2 measurements), group comparisons were performed separately at each time point using one-way ANOVA. The individual mouse was considered the biological replicate (n = 5 per group).

P-values are indicated as follows: *p < 0.05, **p < 0.01, ***p < 0.001, and ****p < 0.0001.

## Results

### *E. histolytica* viability and adaptation to indole exposure

The impact of indole on *E. histolytica* in the human colon on the parasite’s physiology remains largely unexplored. To address this, we evaluated the cytotoxicity of indole on *E. histolytica* trophozoites. The trophozoites were exposed to varying concentrations of indole for 24 hours at 37°C. Our findings indicate that indole affects the viability of *E. histolytica*, with an observed IC_50_ of 1.2 mM after 24 hours (S1A Fig). Additionally, previous studies show that *E. histolytica* can adapt to various physiological challenges and toxic conditions, including glucose starvation, exposure to auranofin, and metronidazole treatment [41–43]. This adaptability suggests that *E. histolytica* might also develop responses to indole-induced stress. To explore this, we investigated the parasite’s capacity to adapt to indole. Trophozoites were progressively exposed to increasing concentrations of indole until reaching 1.2 mM (trophozoites adapted to 1.2 mM indole: ADI strain). At this concentration of indole, the doubling time of the adapted ADI strain takes 18.1 hours, compared to 12.4 hours for the control WT strain cultivated without indole (S1B Fig). Reversion of ADI trophozoites to growth in absence of indole for a month restored a doubling time and indole IC_50_ comparable to WT trophozoites (S1A and S1B Fig).

**Fig 1:**
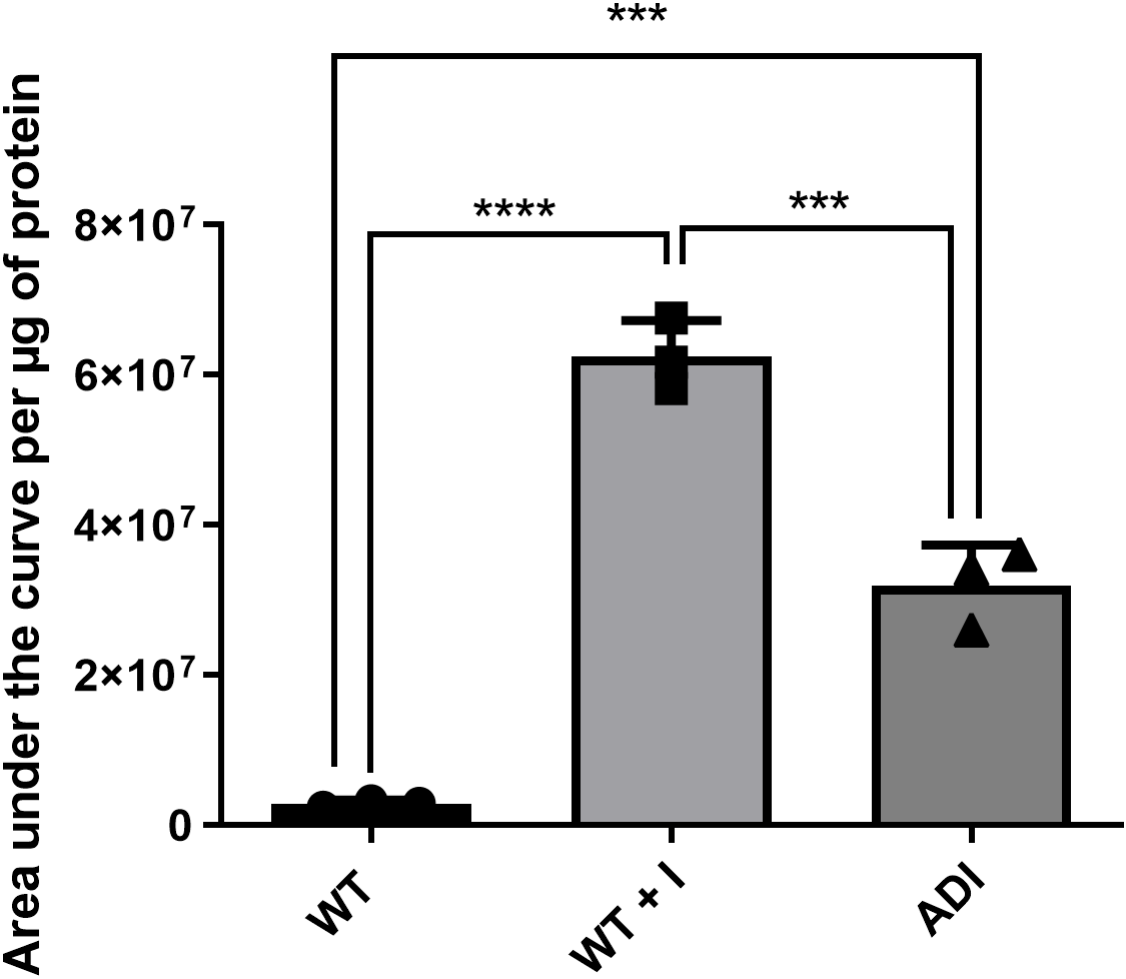
Intracellular indole levels in WT, WT + I, and ADI trophozoites. Indole concentrations in lysates from WT, WT + I, and ADI trophozoites were quantified by liquid chromatography–mass spectrometry (LC–MS) and normalized to total protein content (µg protein). Trace levels of indole were detected in TYI-S-33 medium (1.75 × 10⁴, area under the curve, S1 Data). Statistical analysis was performed using one-way ANOVA. ***P < 0.001; ****P < 0.0001. Data represent the mean of three independent biological replicates.

To understand how indole could cause this toxic effect on trophozoites, the capacity of the indole to penetrate trophozoites was investigated. Indole acts as an intercellular signal in microbial communities [12], as it can cross bacterial lipid membranes, such as those in *E. coli*, without requiring a protein-based transport system, which suggests that it could potentially diffuse through membranes in other biological kingdoms as well [44]. Therefore, we hypothesize that indole may similarly diffuse across the membrane of *E. histolytica* trophozoites and accumulate in their cytoplasm, initiating a process that ultimately leads to their death. To test this hypothesis, we quantified indole levels in *E. histolytica* trophozoites using metabolomics analysis under three different conditions: wild-type trophozoites not exposed to indole (WT), wild-type trophozoites exposed to 1.2 mM indole for 24 hours (WT + I), and trophozoites adapted to 1.2 mM indole (ADI). Intracellular indole levels were significantly higher in both WT + I and ADI trophozoites compared with WT, indicating that indole is taken up into the cytoplasm of *E. histolytica* (Fig 1). Notably, indole abundance was lower in ADI than in WT + I (Fig 1), suggesting that long-term adaptation is associated with reduced intracellular indole accumulation relative to acute exposure. Low levels of indole were also detected in WT trophozoites, consistent with endogenous production from tryptophan via tryptophanase [19]. Trace amounts of indole were present in TYI-S-33 medium (S1 Data, Fig 1).

### Protein expression changes in *E. histolytica* in response to indole

To investigate the effect of indole on *E. histolytica*, we conducted a proteomic analysis comparing protein abundance in WT, WT + I and ADI trophozoites (S1 Table). The heatmap clearly shows a distinction in proteomic profiles, highlighting significant differences between, on one side, WT and WT + I, and the other side, ADI trophozoites (S2A Fig). These findings suggest that trophozoites have a different proteomic response after adaptation to indole. To further explore the molecular basis of this adaptation, we analyzed differential protein expression and categorized the affected proteins using PANTHER, a bioinformatics tool that classifies proteins into functional classes.

**Fig 2:**
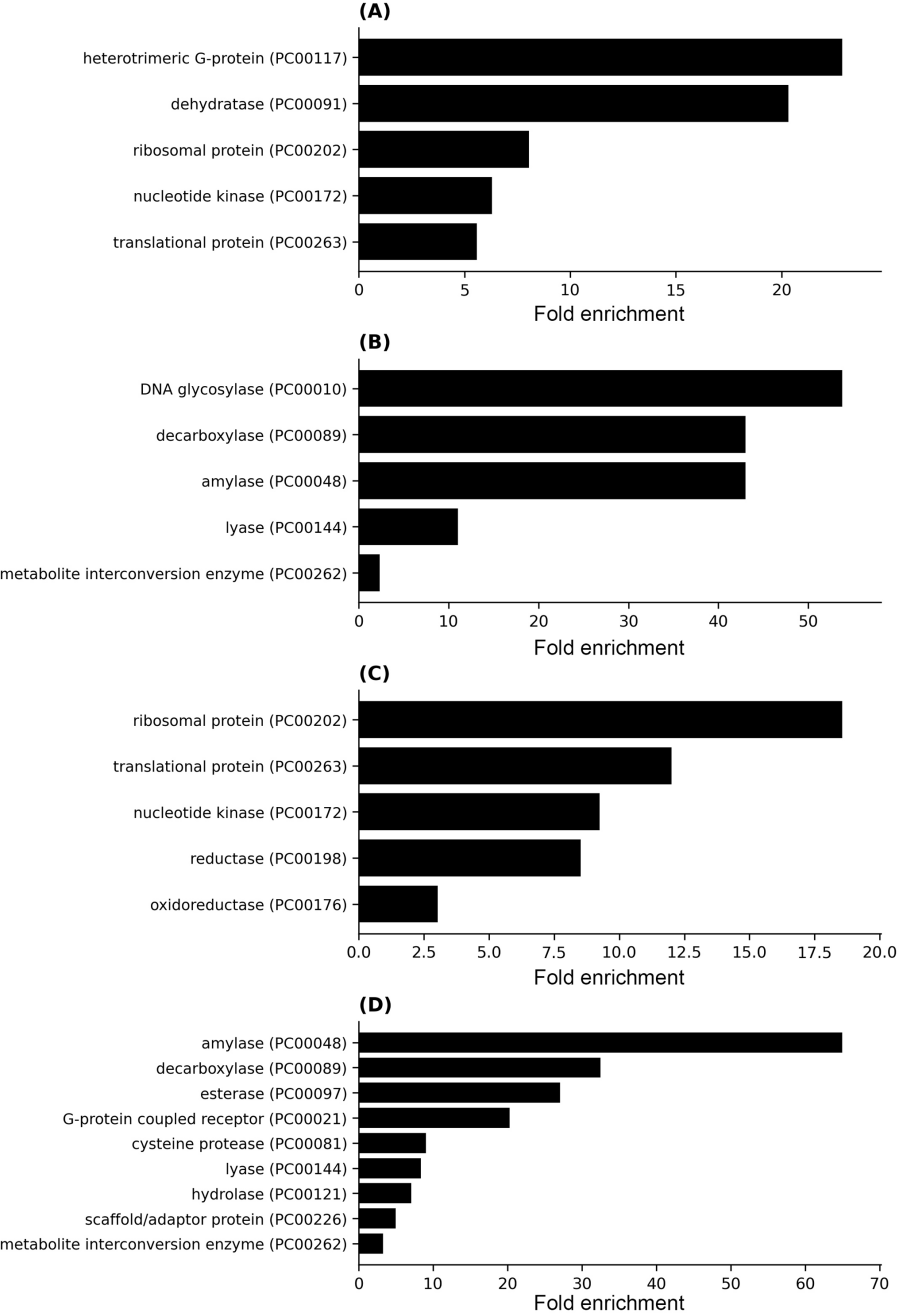
Differential protein class enrichment in WT, WT + I, and ADI trophozoites. (A) Upregulated proteins in ADI compared to WT. (B) Downregulated proteins in ADI compared to WT. (C) Upregulated proteins in ADI compared to WT + I. (D) Downregulated proteins in ADI compared to WT + I.

Upregulated proteins in ADI compared to WT include translational protein (PC00263) and ribosomal protein (PC00202), suggesting an upregulation of protein translation. Cytoskeleton-related proteins are also upregulated in ADI compared to WT, suggesting a more dynamic cytoskeleton (Fig 2A). For instance, cytoskeletal proteins such as actin putative (EHI_043640), protein-binding activity modulator (PC00095), and Rho guanine nucleotide exchange factor (EHI_100140) (S2B Fig, S1 Table). Virulence factors or virulence-related proteins are found to be both upregulated and downregulated in ADI compared to WT. Protein modifying enzymes (PC00260), where cysteine proteinase is the perfect example; four cysteine proteases (EHI_180170, EHI_010580, and EHI_039310) are upregulated, while CP1 is downregulated in ADI compared to WT (S2B Fig, S1 Table). Eukaryotic translation initiation factor 5A (EHI_151540) and Galactose binding lectin 35 kDa subunit (EHI_027800) are upregulated in ADI compared to WT. However, pore-forming peptide amoeba pore B (EHI_194540) is downregulated (S2C Fig, S1 Table).

ADI compared to WT + I, shows similar upregulated proteins to ADI compared to WT. For instance, the upregulation of translational protein (PC00263) such as 40S ribosomal protein (EHI_105180), or 60S ribosomal protein (EHI_152570), cytoskeletal protein (PC00085) such as Actin (EHI_043640) and protein-binding activity modulator (PC00095) such as Rho guanine nucleotide exchange factor, (EHI_110980) (Fig 2C, S2D Fig, S1 Table). In addition, ADI, compared to WT + I, presents an upregulation of oxidoreductase (PC00176) such as malate dehydrogenase (EHI_030810) and chaperone (PC00072), like heat shock protein 70, putative (EHI_150770), suggesting an increased resistance of ADI to OS (Fig 2C, S1 Table). The virulence factor Galactose binding lectin 35 kDa subunit (EHI_027800) is also upregulated in ADI compared to WT + I, (S1 Table). However, other virulence factors are downregulated in ADI compared to WT + I, like cysteine proteinase (PC00081, EHI_151440, EHI_050570, EHI_074180) or pore-forming peptide amoebapore A (EHI_159480), pore-forming peptide amoebapore B (EHI_194540) (Fig 2D, Fig S2E, S1 Table).

### Indole adaptation confers enhanced tolerance to oxidative stress in *E. histolytica*

Proteomics data indicate an upregulation of oxidoreductase proteins, including peroxiredoxin [45], in ADI compared to WT + I, suggesting that redox regulation may contribute to adaptation to indole. Among the oxidoreductase proteins, glyceraldehyde-3-phosphate dehydrogenase (GAPDH; EHI_060860) and glutamate synthase beta subunit (EHI_110520) are upregulated in ADI relative to WT + I (Fig 2C and S1 Table). Based on these findings, we hypothesized that trophozoites adapted to indole would exhibit altered responses to OS.

**Fig 3:**
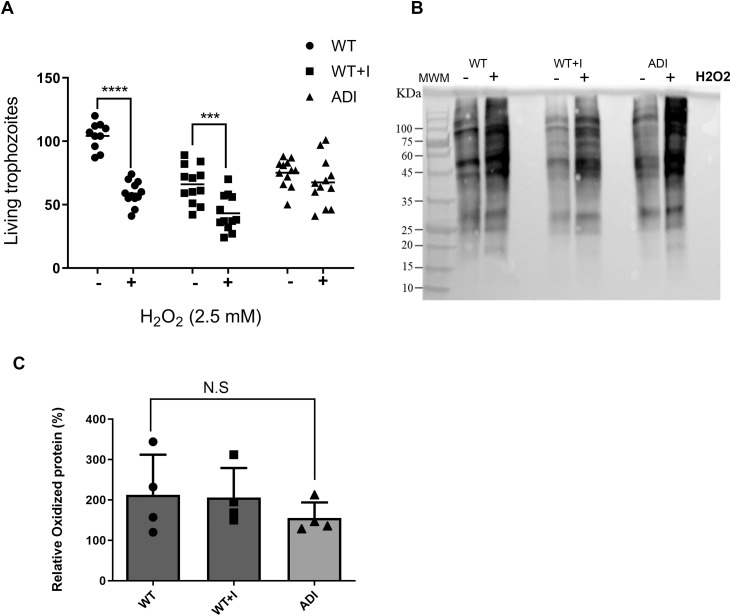
Oxidative stress response and protein oxidation in WT, WT + I, and ADI trophozoites. (A) WT, WT + I, and ADI trophozoites were incubated with 2.5 mM H₂O₂ for 30 min at 37°C. Cell survival was assessed by eosin exclusion assay. (B) Representative OxyBlot detecting protein carbonyl groups in trophozoites cultured in the presence or absence of H₂O₂. (C) Quantification of OxyBlot signals presented as fold change relative to untreated controls. Statistical analysis was performed using two-way ANOVA for (A) and one-way ANOVA for (C). ***p < 0.001; ****p < 0.0001. Data represent the mean of three independent biological replicates for (A) and four independent biological replicates for (B–C).

To test this, trophozoites (WT, WT + I, and ADI) were exposed to H₂O₂ (2.5 mM for 30 minutes), and cell survival was quantified using the eosin exclusion assay. ADI trophozoites retained significantly higher viability following H₂O₂ exposure compared to both WT and WT + I (Fig 3A), indicating enhanced tolerance to oxidative challenge. Although WT + I trophozoites showed lower viability overall, the proportional decrease in survival after H₂O₂ treatment was similar to that observed in WT cells. This suggests that acute indole exposure primarily reduces overall cell health, rather than specifically increasing sensitivity to oxidative stress.

To determine whether the improved survival of ADI trophozoites was associated with reduced oxidative protein damage, we quantified protein carbonylation under basal conditions and after H₂O₂ exposure (Fig 3B). No significant differences in protein carbonyl levels were detected among WT, WT + I, and ADI following oxidative stress (Fig 3C). These findings suggest that the enhanced survival of ADI trophozoites is not due to reduced protein oxidation itself, but may instead reflect improved tolerance to oxidative damage or more efficient recovery mechanisms.

### Effect of indole on protein translation

**Fig 4:**
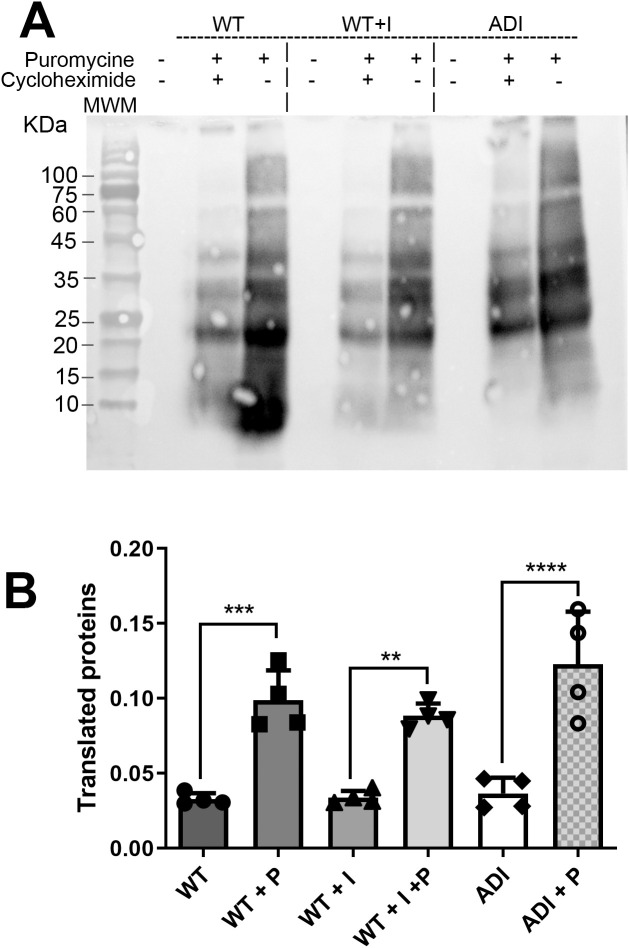
Global protein translation in WT, WT + I, and ADI trophozoites. (A) Representative SUnSET assay showing detection of newly synthesized proteins following puromycin incorporation (10 μg/mL). Cycloheximide (100 μg/mL) was used as a negative control to inhibit translation. (B) Quantification of SUnSET signals. Statistical analysis was performed using one-way ANOVA. ***p < 0.001; ****p < 0.0001. Data represent the mean of four independent biological replicates.

Proteins involved in translation are upregulated in ADI compared to WT and WT + I. Previous studies have shown that protein translation levels increase in response to other stressors, such as heat shock [46]. Notably, the translation initiation factor 5A (eIF5A1, EHI_151540) is significantly upregulated in ADI and is known to play a key role in elongation, termination, and stimulation of peptide bond formation [47]. Additionally, multiple ribosomal subunits show increased expression in ADI compared to WT. These include small (40S) ribosomal proteins (S23, S13, S28, S26, S17, S6, and S18), as well as large (60S) ribosomal proteins (L31, L38, L24, L35a, and L11). Heterotrimeric G-proteins, which are membrane-associated proteins that couple with seven transmembrane receptors to transmit intracellular signals, are also upregulated in ADI. Together, these proteomic changes suggest remodeling of the translational and signaling machinery in ADI trophozoites. To determine whether these changes translate into increased global protein synthesis, we quantified nascent protein production using the SUnSET assay, which measures puromycin incorporation into newly synthesized polypeptides. Although ADI trophozoites showed a trend toward increased puromycin incorporation, the difference was not statistically significant compared to WT and WT + I (Fig 4A and 4B). These results indicate that indole adaptation is associated with altered abundance of translational components without a detectable increase in overall protein synthesis under the conditions tested.

### Increased level of filamentous actin (F-actin) and trophozoite motility in ADI trophozoites

An actin protein, EHI_043640, is upregulated in ADI compared to both WT and WT + I (S1 Table). Interestingly, this upregulated protein is a truncated version of an actin protein (EHI_182900), and it is not clear if this truncated version is a functional protein [48]. Actin plays a crucial role in *E. histolytica*’s ability to migrate, divide, exert cytotoxicity, and in the phagocytosis of human cells [48], particularly in the form of F-actin, which regulates the assembly and disassembly of microfilaments in amoeba [48]. Other cytoskeleton-related proteins were found upregulated, like α-actinins (EHI_161200), involved in amoeboid motility and phagocytosis, as well as regulators of actin dynamics [49], such as RhoGAPs and RhoGEFs, which collectively support active cytoskeletal remodeling in ADI.

**Fig 5:**
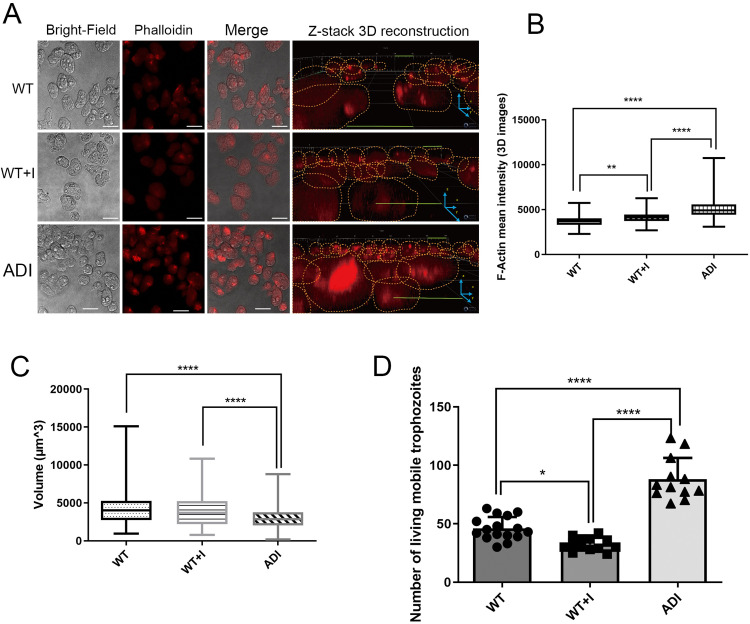
F-actin levels, cell size, and motility in WT, WT + I, and ADI trophozoites. (A) Representative confocal microscopy images (20×) of trophozoites stained with phalloidin (red) to visualize F-actin. Scale bar, 30 µm. (B) Quantification of F-actin intensity from three-dimensional reconstructions using Imaris software. (C) Cell volume measurements derived from three-dimensional confocal reconstructions. Phalloidin staining was performed in duplicate within each independent experiment. (D) Motility assay showing trophozoites migrating through an 8 µm pore membrane in Transwell assays. Experiments were performed in triplicate. Statistical analysis was performed using one-way ANOVA. *p < 0.05; ****p < 0.0001. Data represent the mean of two independent biological replicates.

To determine if the increase in cytoskeletal proteins observed in ADI has an effect on F-actin formation, we compared the level of F-actin in WT, WT + I, and ADI trophozoites (Fig 5A and S1 Table). First, two-dimensional microscopy images and three-dimensional z-stack images were taken with fluorescent confocal microscopy and were analyzed, respectively, with the Cellpose-SAM model coupled with Fiji and Imaris software. Results indicate that F-actin formation measured by fluorescent intensity was increased in ADI compared to WT and WT + I (Figs 5B and S3A). The amount of F-actin was also increased in WT + I relative to WT (Figs 5B and S3A). Interestingly, ADI are smaller in size (lower cell area and cell volume) than WT or WT + I (Figs 5C and S3C).

To directly assess trophozoite motility, a transwell migration assay was conducted. This assay quantifies the ability of trophozoites to migrate through 8 μm pores in a membrane, thereby assessing their motility under different conditions (WT, WT + I, and ADI). The mobility in ADI increased by 42% compared to WT (Fig 5D). Although WT + I cells exhibited an equivalent cell volume to WT cells (Fig 5C), fewer WT + I cells migrated to the lower chamber compared to WT (Fig 5D).

### Indole adaptation reduces cytopathic activity and modulates intestinal inflammation while enhancing cecal colonization in mice

**Fig 6:**
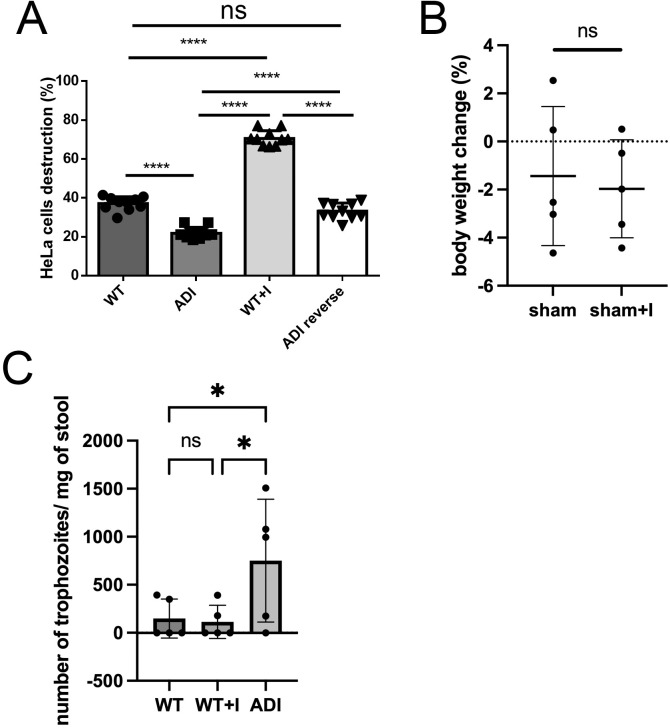
Cytopathic activity and in vivo colonization of WT, WT + I, and ADI trophozoites. (A) WT, WT + I, ADI, and ADI-reverse trophozoites (3 × 10⁵ cells) were co-incubated with HeLa cells for 30 min. Surviving HeLa cells were quantified by methylene blue staining. Data represent the mean of two independent biological replicates. (B) Change in mouse body weight at day 7 in sham-operated controls and mice inoculated with 1.2 mM indole (n = 5 per group). (C) Parasite burden quantified seven days after intracecal injection of trophozoites cultured in the presence (WT + I, ADI) or absence (WT) of 1.2 mM indole. Statistical analysis was performed using one-way ANOVA followed by Fisher’s LSD post hoc test. *p < 0.05.

Indole negatively affects virulence traits in bacteria such as *Listeria monocytogenes* [50] and *Vibrio tasmaniensis* [51], as well as in protozoa like *Cryptosporidium* [22]. To assess the impact of indole on *E. histolytica* fitness, we measured cytopathic activity on HeLa cells and the ability to colonize the mouse cecum. WT + I trophozoites exhibited a two-fold increase in cytopathic activity compared to WT trophozoites, whereas ADI trophozoites showed a two-fold decrease relative to WT (Fig 6A). ADI reverse trophozoites displayed cytopathic activity comparable to WT (Fig 6A), indicating that the phenotype is reversible and dependent on sustained indole exposure.

We next evaluated parasite colonization in a mouse model of amebic colitis. *E. histolytica* establishes intestinal infection following intracecal injection [9]. WT, WT + I, and ADI trophozoites were injected into the mouse cecum, and colonization was assessed seven days post-infection by quantifying *E. histolytica* 18S rRNA in stool samples. Inoculation with 1.2 mM indole did not result in significant weight loss compared with sham-operated controls after day 7 in mice (n = 5 per group) (Fig 6B). Seven days post-infection, the ADI strain colonized the cecum at levels approximately twofold higher than those of the WT and WT + I groups, as determined by quantification of Entamoeba-specific rRNA gene copies in stool samples (Fig 6C). In contrast, WT + I trophozoites did not exhibit increased colonization compared with WT.

**Fig 7:**
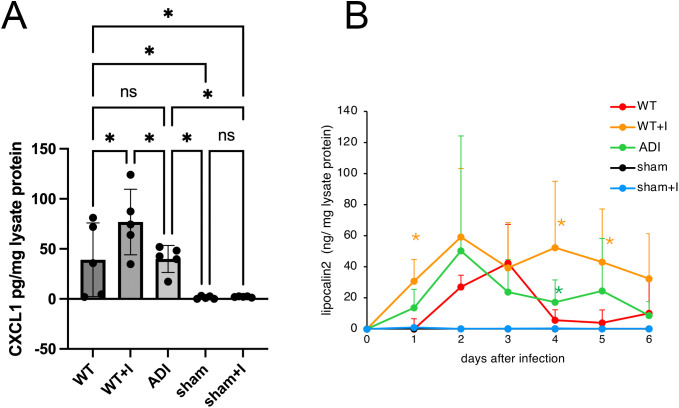
Intestinal inflammatory responses to WT, WT+I, and ADI trophozoite infection. (A) CXCL1 levels in cecal tissue 24 h after intracecal injection of trophozoites cultured in the presence (WT+I, ADI) or absence (WT) of 1.2 mM indole, as well as sham-operated controls with or without indole. (B) Fecal lipocalin-2 levels measured daily for up to 6 days post-infection by ELISA. Statistical analysis was performed separately at each time point using one-way ANOVA followed by Fisher’s LSD post hoc test. *p < 0.05. n = 5 mice per group.

To assess host inflammatory responses, we measured CXCL1, a neutrophil-recruiting chemokine [52], and fecal lipocalin-2, a marker of intestinal inflammation [53]. CXCL1 was undetectable 24 hours after injection in sham-operated and 1.2 mM indole-injected control groups (Fig 7A). In contrast, CXCL1 expression was induced specifically in response to *Entamoeba* infection. The WT + I group exhibited the highest CXCL1 levels, whereas the WT and ADI groups showed comparable expression (Fig 7A).

Fecal lipocalin-2 levels were measured daily for six days following infection. Consistent with the CXCL1 results, lipocalin-2 was not detectable in sham-operated or indole-injected control groups during this period (Fig 7B). In the WT group, lipocalin-2 levels peaked on day 3 and declined beginning on day 4. The ADI strain similarly induced lipocalin-2 expression, with a peak on day 2 followed by a decline. In contrast, the WT + I group exhibited higher lipocalin-2 levels than the WT group on days 4 and 5.

## Discussion

Although *Entamoeba histolytica* is routinely studied under axenic conditions, these experimental systems may not fully recapitulate the intestinal environment, where the parasite is exposed to a diverse and dynamic repertoire of microbiota-derived metabolites. Among these, indole reaches millimolar concentrations in the colon and plays well-established roles in microbial communication and host immune regulation [16]. In this study, we show that indole exerts different effects on *E. histolytica*, acting initially as a potent environmental stressor but, upon long-term exposure, as an ecological signal that drives adaptive reprogramming of parasite physiology toward enhanced colonization capacity within the host intestine.

Acute exposure to indole strongly inhibits trophozoite growth, with an IC₅₀ of 1.2 mM, within physiologically relevant concentrations reported in the gut [14,15]. Metabolomic analyses demonstrate intracellular indole accumulation, consistent with passive diffusion across biological membranes without the need for a specific transporter [44,54]. This intracellular accumulation correlates with reduced parasite viability, supporting indole as a genuine environmental challenge for *E. histolytica* rather than a neutral metabolic byproduct. Importantly, trophozoites acutely exposed to indole trigger an enhanced inflammatory response in vivo, characterized by increased CXCL1 and fecal lipocalin levels, suggesting compromised host–parasite compatibility. Proteomic analysis revealed that acute indole exposure increases the abundance of cysteine proteases EhCP1, EhCP4, and EhCP6, which are less abundant in indole-adapted trophozoites. EhCP1, more abundant in WT + I, has been shown to be present in secretory product (SP) of *E. histolytica*. The presence of EhCP1 in the SP stimulates mast cells to produce IL-8, which mediates tissue inflammatory responses during the early phase of human amoebiasis [56]. EhCP4, also more highly expressed in WT + I, is secreted by the parasite and increases inflammation in the mouse cecum [55]. These findings suggest that the elevation of these specific CPs in WT + I contributes to epithelial damage, as supported by cytopathic activity data, and promotes the release of damage-associated molecular patterns and activation of host inflammatory pathways, consistent with exacerbated intestinal inflammation [56–58].

In contrast, progressive adaptation to indole profoundly reshapes *E. histolytica* biology. Although ADI trophozoites accumulate less intracellular indole than acutely exposed WT + I parasites, they tolerate sustained indole exposure and display enhanced resistance to OS, marked cytoskeletal remodeling, increased motility, and improved survival and colonization in the mouse cecum. Reduced intracellular indole levels in ADI may reflect adaptive changes that limit indole accumulation during long-term exposure, thereby contributing to increased resistance. Proteomic analyses reveal coordinated changes in actin dynamics, redox homeostasis, and proteostasis, consistent with a global adaptive program rather than activation of a single pathway. Functionally, these changes translate into enhanced colonization in vivo accompanied by a more restrained inflammatory response compared with acutely exposed trophozoites. WT + I infection was associated with elevated CXCL1 and lipocalin-2 levels, whereas ADI-infected mice exhibited reduced inflammatory markers and kinetics more comparable to WT infection. Whether this attenuated inflammation directly facilitates colonization or instead reflects intrinsic alterations in parasite physiology remains to be determined. In many host–parasite systems, successful persistence depends on immunomodulatory strategies that limit host inflammation while permitting colonization [59,60]. *E. histolytica* similarly deploys factors that modulate host immunity, reduce IFNγ responses, neutralize immune mediators, and adapt metabolism to withstand reactive oxygen and nitrogen species [61]. Indole adaptation may therefore represent an environmentally driven shift toward a host-compatible phenotype consistent with asymptomatic intestinal carriage. Importantly, persistence in the intestine also depends on how trophozoites exploit host resources such as the mucus layer. β-amylases involved in mucus degradation [62] are reduced in ADI compared to WT + I. Interestingly, β-amylase expression is known to be suppressed in the presence of bacteria [63], suggesting that indole may function as a microbial cue reflecting bacterial abundance and thereby reducing reliance on amylase-dependent nutrient acquisition.

Cytoskeletal remodeling emerges as a central feature of indole adaptation. ADI trophozoites exhibit enhanced motility that correlates with increased F-actin levels and the upregulation of actin-associated proteins, including α-actinin-2, EHI_161200, regulators of Rho GTPase signalling (RhoGEFs and RhoGAPs), and the actin protein EHI_043640. Actin filaments form dynamic structures that shape the cell and drive directional movement, enabling the parasite to navigate throughout the tissue and its dissemination within the gut [48]. α-Actinin-2 stabilizes the cytoskeleton by crosslinking actin filaments [64], while RhoGEFs activate Rho GTPases to promote actin polymerization and structure formation, and RhoGAPs inactivate them to trigger filament disassembly, together orchestrating dynamic cytoskeletal regulation [65]. Notably, EHI_043640 is a truncated version of EHI_182900 with an identical amino acid sequence. Its selective upregulation under indole exposure, in ADI trophozoites, and during human colon invasion [66] suggests a role in cytoskeletal plasticity that may facilitate motility, host tissue penetration, and adaptation to gut environmental cues.

In addition to enhanced cytoskeletal activity, ADI trophozoites display a reduced cell size, a physical property likely contributing to enhanced amoeboid motility. For low traction forces and limited frictional drag migration, smaller cell bodies facilitate faster and more efficient movement through dense and heterogeneous environments, such as the intestinal mucus layer or host tissues [67]. Similar relationships between reduced cell size, increased motility, and diminished contractile forces have been described in other biological systems, including transformed epithelial cells [68]. Thus, indole adaptation appears to link cytoskeletal remodelling with biophysical optimization of parasite movement. Finally, the enhanced cytoskeletal activity in ADI trophozoites likely underlies their improved colonization capacity, which is also heavily reliant on cytoskeletal function [69].

Resistance to oxidative stress is another defining feature of indole adaptation. ADI trophozoites survive oxidative challenge more efficiently than WT or acutely exposed parasites, consistent with the increased abundance of oxidoreductases and other oxidative stress–response proteins. Importantly, levels of oxidized proteins following H₂O₂ exposure remain comparable across conditions, indicating that indole adaptation does not necessarily reduce the extent of protein oxidation. Rather, these findings suggest that ADI trophozoites exhibit enhanced tolerance to oxidative damage, likely through improved management of damaged proteins. Elevated expression of chaperones such as Hsp70 supports a model in which oxidatively modified proteins are more efficiently refolded or degraded, allowing ADI trophozoites to better preserve cellular function under stress [70].

Although several components of the translational machinery are upregulated in ADI trophozoites, global protein synthesis remains unchanged. The SUnSET assay revealed no significant differences in overall translation between ADI, WT, and WT + I strains. These observations indicate that indole adaptation is associated with remodeling of translational components rather than a generalized increase in protein production. Such remodeling may support selective synthesis or turnover of stress-response proteins, contributing to improved proteostasis without altering total translational output. Together, the coordinated enrichment of translational machinery, oxidoreductases, and chaperones suggests that indole adaptation promotes an integrated reorganization of proteostasis pathways that enhances cellular resilience to oxidative stress.

**Fig 8:**
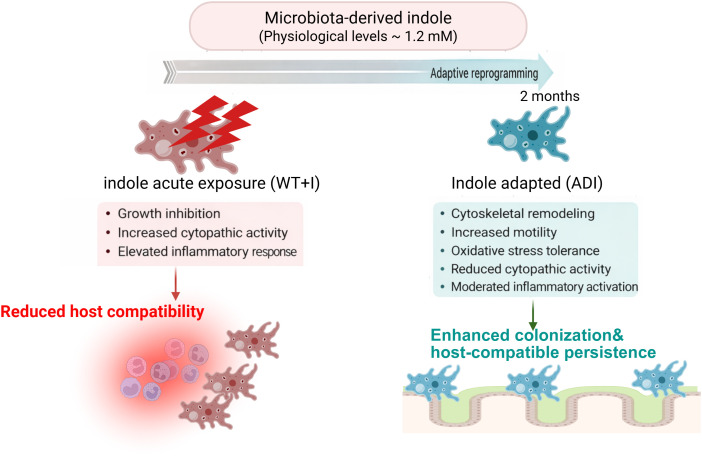
Model of indole-driven adaptive reprogramming in E. histolytica. Schematic representation of the proposed model in which microbiota-derived indole differentially influences *E. histolytica* physiology depending on exposure duration. Acute exposure to physiological concentrations of indole imposes metabolic stress, reduces trophozoite growth, increases cytopathic activity, and enhances host inflammatory responses. In contrast, sustained exposure promotes adaptive reprogramming characterized by cytoskeletal remodeling, reduced cell size, enhanced motility, improved oxidative stress tolerance, moderated inflammatory activation, and increased intestinal colonization. Together, these findings support a model in which indole functions as an environmental cue that reshapes parasite physiology toward a host-compatible, persistence-oriented phenotype within the intestinal niche. Created in BioRender. Ankri, S. (2026) https://BioRender.com/04bmdql.

Taken together, our data (summarized in Fig 8) support a model in which microbiota-derived indole initially imposes stress on *E. histolytica* but, upon sustained exposure, drives adaptive reprogramming toward enhanced motility, oxidative stress tolerance, and host-compatible colonization. These findings underscore the importance of incorporating gut-relevant environmental cues into experimental models and highlight the remarkable plasticity of *E. histolytica* in integrating microbial signals to optimize survival and intestinal colonization within the host. More broadly, they suggest that microbiota-derived metabolites should be viewed not only as modulators of host immunity but also as key drivers of parasite physiology and disease outcome in amoebiasis.

## Supporting information

S1 Table Proteomics data showing all proteins up and down regulated in trophozoites (WT, WT + I, and ADI) detected by mass spectroscopy. The proteins were cleaved with trypsin and analyzed by the LC/MSMS using the Exploris480 (Thermo) mass spectrometer in DIA mode in order to increase the number of identifications. The data was analyzed using DIA-NN 1.9.1 for identification and quantification against *E. histolytica* proteome from UniProt with 1% FDR threshold (FDR = false discovery rate, is the estimated fraction of false positives in a list of peptides). Additional statistical analysis was done by Perseus 1.6.7.0. The protein list contains the quantitation values, peptide count, T-test and annotations columns. The significantly changed proteins are marked as detailed in the color legend on the top rows. Difference is log 2 (fold changed), Log2(0) in log2 intensity columns was replaced with 13 (the threshold intensity in the project). A protein identified with a single peptide could not be considered as a certain identification marked in red in column called “# of peptides” (column AF).
(XLSX)

S1 Fig Indole sensitivity and growth kinetics of WT, ADI, and ADI-reverse trophozoites. (A) IC₅₀ of indole for WT and ADI-reverse trophozoites. (B) Doubling time determined from trophozoite counts at 0 and 24 h. Statistical analysis was performed using one-way ANOVA. ****p < 0.0001.
(TIF)

S2 Fig Proteomic profiling of WT, WT + I, and ADI trophozoites. (A) Heatmap of proteomics results (n = 3). (B–E) Differentially regulated protein classes as indicated. (B) Upregulated in ADI compared to WT, (C) Downregulated in ADI compared to WT, (D) Upregulated in ADI compared to WT+I, (E) Downregulated in ADI compared to WT+I.
(TIF)

S3 Fig Two-dimensional analysis of F-actin levels and cell size in WT, WT + I, and ADI trophozoites. (A) Quantification of F-actin intensity from two-dimensional confocal images. (B) Quantification of cell area. Statistical analysis was performed using one-way ANOVA. P < 0.05; ****P < 0.0001. Data represent the mean of two independent biological replicates.
(TIF)

S1 Data Raw dataset underlying the results presented in this study. (ZIP)
