# Supplementary figures and images for: Microbiota-derived indole differentially shapes Entamoeba histolytica physiology and promotes host-compatible colonization
Source: PLoS Negl Trop Dis. 2026 Apr 13;20(4):e0013416. doi: 10.1371/journal.pntd.0013416 (PMC13095123; doi:10.1371/journal.pntd.0013416)

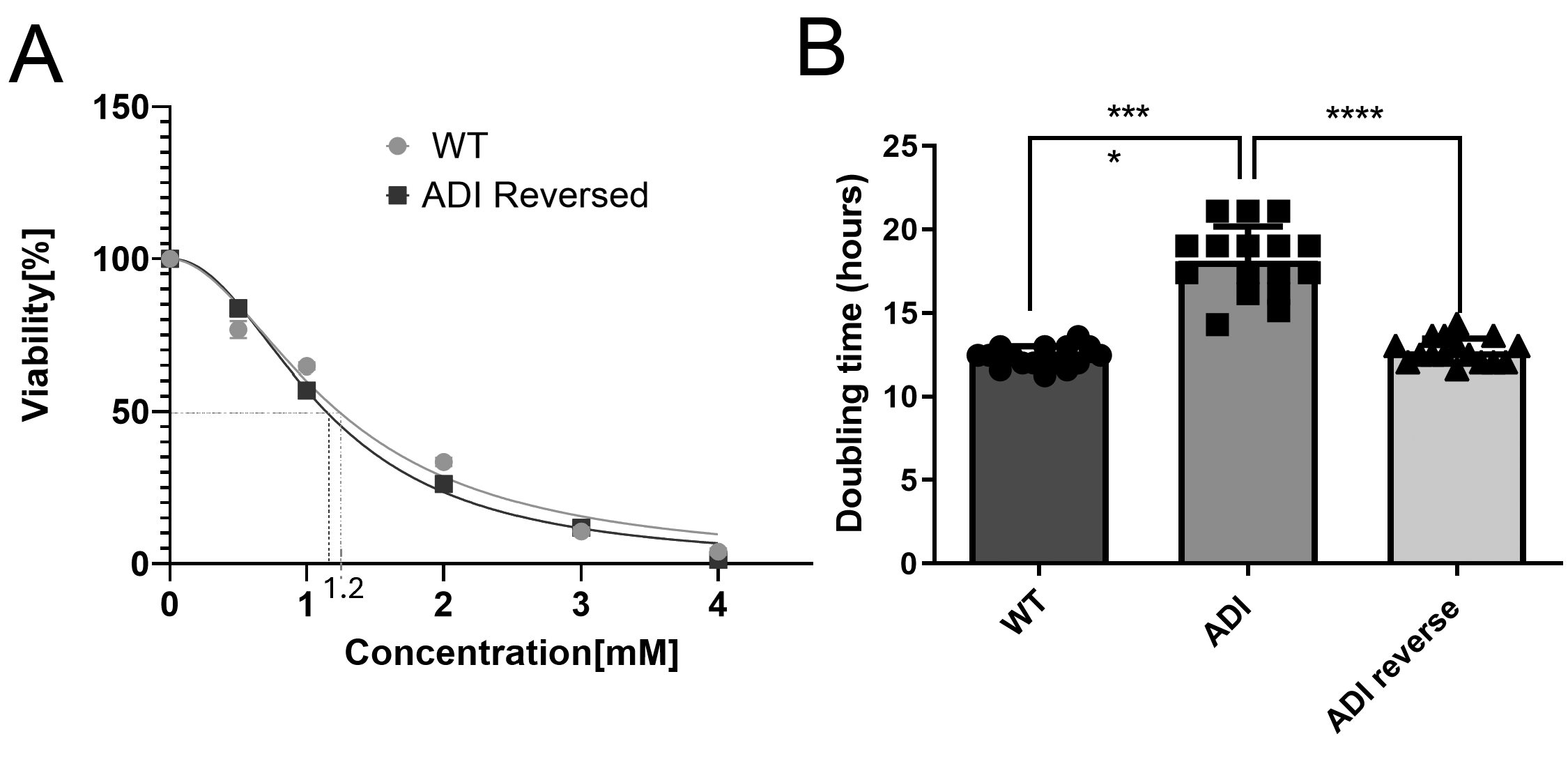

Supplement: S1 Fig — (A) IC₅₀ of indole for WT and ADI-reverse trophozoites. (B) Doubling time determined from trophozoite counts at 0 and 24 h. Statistical analysis was performed using one-way ANOVA. ****p < 0.0001. (TIF) [file pntd.0013416.s002.tif]

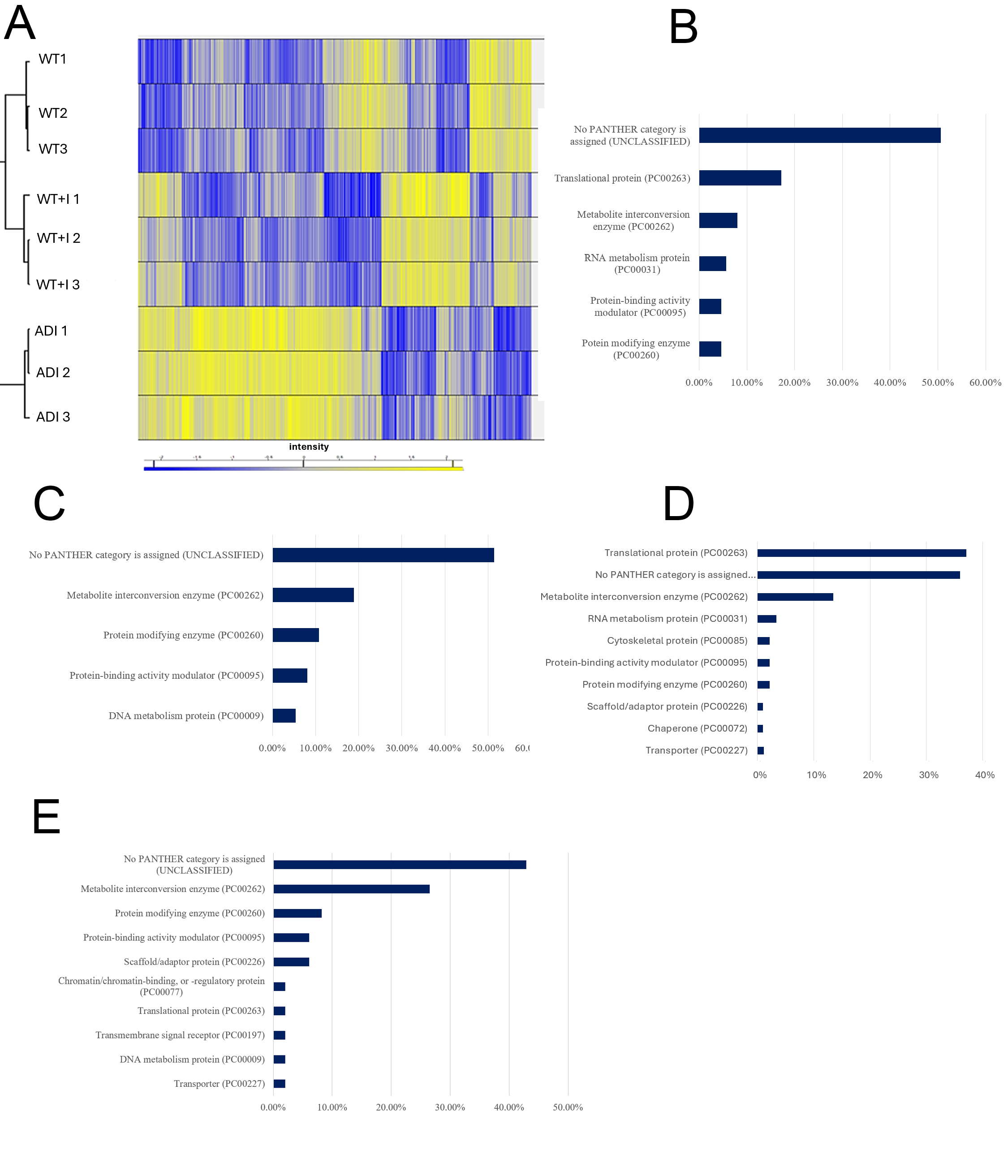

Supplement: S2 Fig — (A) Heatmap of proteomics results (n = 3). (B–E) Differentially regulated protein classes as indicated. (B) Upregulated in ADI compared to WT, (C) Downregulated in ADI compared to WT, (D) Upregulated in ADI compared to WT+I, (E) Downregulated in ADI compared to WT+I. (TIF) [file pntd.0013416.s003.tif]

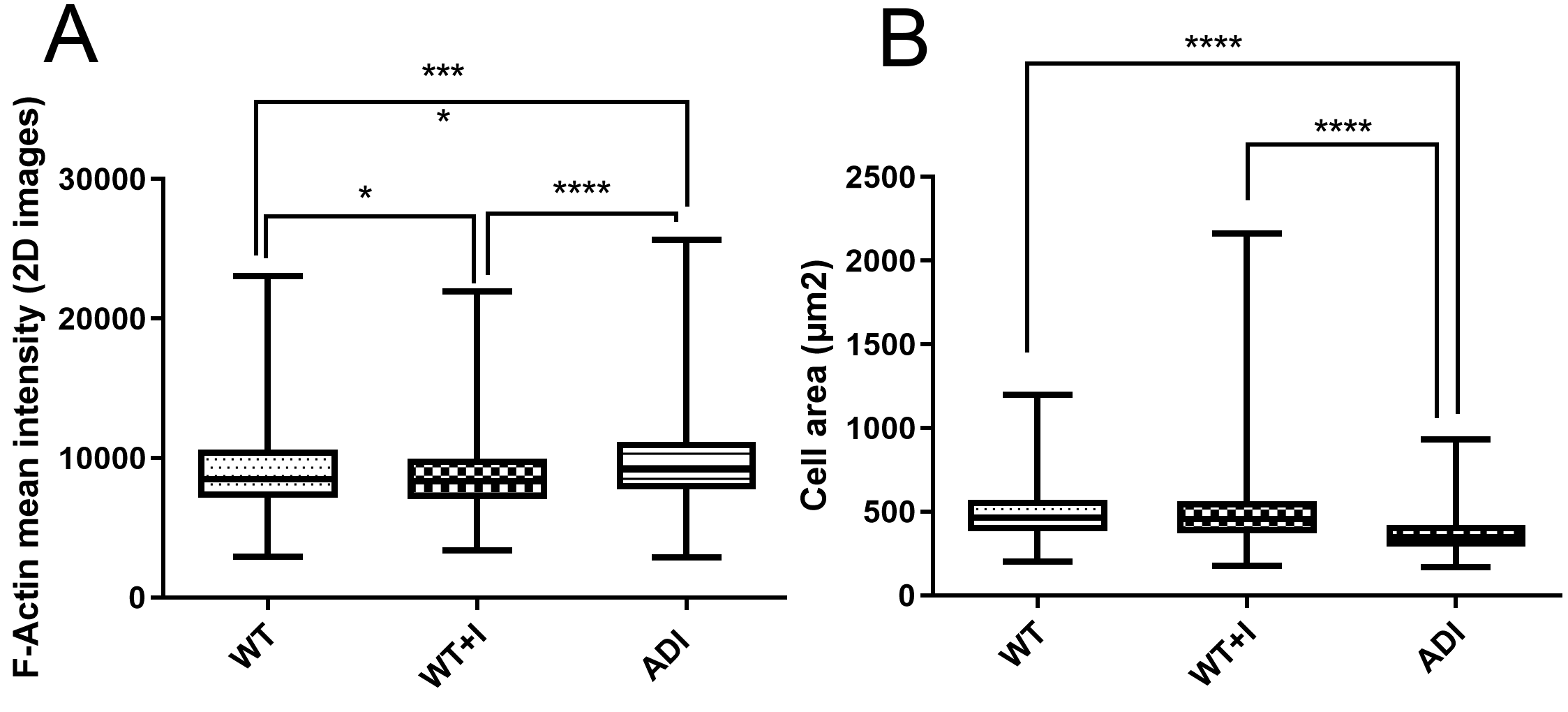

Supplement: S3 Fig — (A) Quantification of F-actin intensity from two-dimensional confocal images. (B) Quantification of cell area. Statistical analysis was performed using one-way ANOVA. P < 0.05; ****P < 0.0001. Data represent the mean of two independent biological replicates. (TIF) [file pntd.0013416.s004.tif]
